# Prefrontal BDNF Levels After Anodal Epidural Direct Current Stimulation in Rats

**DOI:** 10.3389/fphar.2018.00755

**Published:** 2018-07-12

**Authors:** Juliana C. de Souza Custódio, Cleciane W. Martins, Marcelo D. M. V. Lugon, Lívia C. de Melo Rodrigues, Suely G. de Figueiredo, Ester M. Nakamura-Palacios

**Affiliations:** ^1^Laboratory of Cognitive Sciences and Neuropsychopharmacology, Program of Post-Graduation in Physiological Sciences, Federal University of Espírito Santo, Vitória, Brazil; ^2^Laboratory of Chemistry of Proteins, Program of Post-Graduation in Physiological Sciences, Federal University of Espírito Santo, Vitória, Brazil; ^3^Laboratory of Neuroendocrinology and Perinatal Stress, Program of Post-Graduation in Biochemistry and Pharmacology, Federal University of Espírito Santo, Vitória, Brazil; ^4^Laboratory of Neurotoxicology and Psychopharmacology, Program of Post-Graduation in Physiological Sciences, Federal University of Espírito Santo, Vitória, Brazil

**Keywords:** epidural direct current stimulation, prefrontal cortex, brain-derived neurotrophin – BDNF, proBDNF, mBDNF

## Abstract

This study measured levels of brain-derived neurotrophic factor (BDNF) in the prefrontal cortex (PFC) after single (S) and repetitive (R) anodal epidural DC stimulation (eDCS) over the left medial prefrontal cortex (mPFC). Male Wistar rats (*n* = 4 per group) received single application of sham (S-sham) or anodal eDCS (S-eDCS) (400 μA for 11 min) and had their PFC removed 15, 30, or 60 min later. For repetitive brain stimulation, rats received sham (R-sham) or anodal eDCS (R-eDCS) once a day, five consecutive days, and their PFC were removed 24 h after the last application. BDNF isoforms levels were measured by Western blot assays. It was observed that animals receiving S-eDCS showed smaller (*p* < 0.01) levels of BDNF 15 min after stimulation when compared to S-sham, especially in its mature form (mBDNF *p* < 0.001). Levels of BDNF, including mBDNF, were almost like the S-sham at 30 and 60 min intervals after stimulation, but not proBDNF, which was significantly smaller (*p* < 0.05) than S-sham at these intervals. After five sessions, BDNF levels were higher in the PFC of R-eDCS animals, notably the proBDNF (*p* < 0.01) when compared to R-sham. This study showed that levels of BDNF in the PFC, especially the proBDNF, were lower after a single and higher after repetitive anodal eDCS applied over the left mPFC when compared to sham. Therefore, changes of prefrontal BDNF levels may disclose molecular changes underlying the plasticity induced by cortical anodal DC stimulation, which may be opposite if applied in single or multiple sessions.

## Introduction

In the last decade, the transcranial direct current stimulation (tDCS) arose as a useful therapeutic tool for neuropsychiatric disorders (Kuo et al., 2014). It has also shown to improve working memory performance when anodal current is applied over the left dorsolateral prefrontal cortex (DLPFC) in healthy volunteers (Fregni et al., 2005; Hill et al., 2016; Mancuso et al., 2016). In experimental settings, anodal polarization of monkeys’ DLPFC facilitated delayed response learning (Rosen and Stamm, 1972). In rats, the anodal epidural direct current stimulation (eDCS) applied over the left medial Prefrontal Cortex (mPFC) improved long-term spatial working memory (de Souza Custodio et al., 2013).

These studies raised the potential cognitive and clinical beneficial effects of the cortical direct current (DC) stimulation. However, the underlying mechanisms, especially at the molecular level, remain largely unknown. The underlying mechanisms involved in the long-lasting changes induced by DC stimulation of the human motor cortex (Puri et al., 2015) may include the strengthening of synaptic transmission, which is frequently caused by changes in synaptic architecture and expression of brain-derived neurotrophic factor (BDNF).

Brain-derived neurotrophic factor is a well-known member of the neurotrophin family of proteins. In the mature brain, BDNF has been implicated in activity-dependent long-term synaptic plasticity, such as long-term potentiation (LTP), in the hippocampus and neocortex (Lessmann et al., 2003), and consequently in learning and memory processes (Nagappan and Lu, 2005; Greenberg et al., 2009).

This study explored the expression of BDNF – both precursor (proBDNF) and mature (mBDNF) isoforms – in rats’ prefrontal cortex (PFC) at different time intervals after one single session of eDCS (400 μA for 11 min) (Experiment 1) or after its repetitive application (Experiment 2) over the left mPFC.

## Materials and Methods

### Animals

Twenty-four male Wistar rats from our breeding colony (Program of Post-Graduation in Physiological Sciences, Federal University of Espírito Santo, Vitória, Brazil), weighing 250–300 g, were housed individually in a temperature-controlled (20–22°C) room and maintained on a 12-h light/dark cycle, with food and water *ad libitum*. The use of animals followed the ethical principles of the Guide for Care and Use of Laboratory Animals (Garber et al., 2011) and the “EC Directive 86/609/EEC and the Brazilian College of Animal Experimentation (COBEA^1^), which conform to the international principles of research involving animals. This study was approved by the Ethics Committee on Animal Experimentation, Federal University of Espírito Santo (UFES-CEUA 09/2011).

### Electrode Implantation

Rats anesthetized with ketamine (75 mg/kg, IP) and xylazine (10 mg/kg, IP) had their heads fixed in a stereotactic apparatus (David Kopft Instruments, United States), and the skull over the mPFC (*B* = +2.5 mm AP, +1.0 mm L) was abraded over a 5.0 mm circular area with a 1.0-mm drill until the exposition of the dura mater. A round electrode, cut by 5.0 mm diameter from ValuTrode^®^ electrodes (Axelgaard Manufacturing Co., Ltd., United States), was placed on the top of this area filled with conductive gel for EEG and sealed with dental acrylic. The skin over the back of the right side of the head and neck was shaved to allow the attachment of the removable cathode electrode (10.0-mm round) during direct current stimulation.

### Direct Current (DC) Stimulation

Anodal eDCS (400 μA, current density of 20.37 μA/mm^2^) was delivered for 11 min by a constant current stimulator (Model EFF-341, Insight Equipamentos Ltda., Brazil). The animal was awake and handled manually by an experimenter during the stimulation. The removable cathode electrode was attached with tape over the skin. Same procedures were done in the sham stimulation, except that the stimulator was turned off during the experiment.

### Tissue Sampling

After *eDCS* stimulation or *sham* procedure, rats were decapitated using a guillotine (Insight^®^) and had their brains excised and washed with ice-cold saline solution before being blotted dry. Then the tissue corresponding to the PFC was removed over a cooled surface, immersed in liquid nitrogen and kept at -80°C until use.

### Protein Extraction

Tissue samples (≈100 mg) from PFC of control and eDCS-rats were homogenized by sonication (three cycles 15 s with intervals 30 s) (Model TE-099, Marconi Equipamentos para Laboratórios Ltda, Brazil) on ice in 10 volumes of cold lysis buffer (NaCl 137 mM, Tris-HCl 20mM, 0.5 mM sodium vanadate, 10% glycerol, 1% NP40 and 1% protease inhibitor cocktails [Sigma^®^]). Further, the homogenates were centrifuged (10,000 ×*g* for 30 min at 4°C) and the supernatants were collected and assayed for protein quantification (2-D Quant Kit – GE Healthcare^®^). Aliquots of the protein extracts (Rong and Baudry, 1996) were separated into single-use samples and stored at -80°C until Western blotting analysis.

### Western Blot Assay

Briefly, 60 μg of protein extract of PFC from four animals of each group were resolved in a denaturing SDS polyacrylamide gel – 15% (Laemmli, 1970) and colorburst^TM^ electrophoresis markers (Sigma^®^) were used as molecular mass standards. The proteins were transferred onto nitrocellulose membranes (Hybond, Amersham, United Kingdom), using a *trans*-blot wet transfer unit (Bio-Rad^®^), by applying a current of 100 V at 4°C overnight.

Next, the membranes were rinsed with TBS-Tween 0.1% and incubated with blocking buffer (5% low-fat milk powder in TBS-Tween 0.1%) at room temperature for 2 h. *Trans*-blotted proteins were then probed overnight at 4°C with a polyclonal anti-primary antibody directed against BDNF [1:500 (Alomone^®^)] and a polyclonal anti-GAPDH [1:2000 (Santa Cruz)]. After washing three times in TBS-Tween 0.1% for 10 min, the membranes were incubated with horseradish peroxidase (HRP)-conjugated secondary antibody (1: 2,500 dilution) in blocking solution for 1 h.

Specific binding was revealed with a Western blotting detection ECL system (Amersham, United Kingdom) and exposed to a CCD camera (ChemiDoc, Bio-Rad^®^). ProBDNF, mBDNF, and GAPDH signals were processed by Image Lab Software (Bio-Rad^®^) and the signal intensities were measured in delimitated areas of equal size and expressed as arbitrary units. Protein levels of GAPDH were used to normalize the results.

### Statistical Analysis

Data were expressed as means ± SEM (standard error of the mean). In Experiment 1, one-way analysis of variance (ANOVA) for independent measures followed by Bonferroni’s multiple comparisons test was used to compare the BDNF levels (total BDNF, proBDNF or mBDNF) measured after S-sham or S-eDCS at 15, 30, or 60 min. In Experiment 2, BDNF levels after five consecutive sessions were compared between R-sham and R-eDCS groups by unpaired *t*-test. A two-tailed α-level of 0.05 was considered statistically significant. GraphPad Prism 7.0 (GraphPad Software Inc., United States) was used for the statistical analysis and graphic presentation.

## Results

### Experiment 1

**FIGURE 1:**
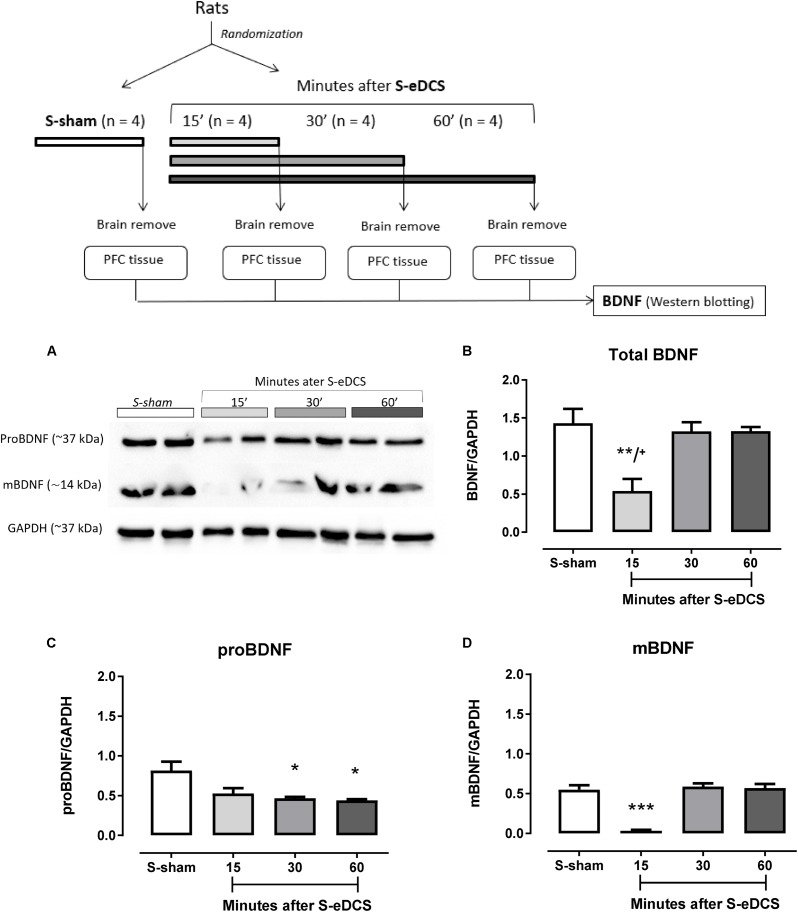
Western blot analysis of brain-derived neurotrophic factor (BDNF) in the prefrontal cortex (PFC) 15 (*n* = 4), 30 (*n* = 4) or 60 (*n* = 4) minutes after a single session of epidural direct current stimulation (S-eDCS) at 400 μA current intensity during 11 min through a 5-mm round electrode implanted over the left medial Prefrontal Cortex (mPFC) or sham procedure (S-sham). A diagram of general procedures is shown at the top. **(A)** Bands of the immunoreactivity related to the migration of the proBDNF and mBDNF shown in two animals – biological replicate – from each group. Densitometry analysis: **(B)** Total BDNF (intensity of proBDNF plus mBDNF), **(C)** proBDNF, and, **(D)** mBDNF. Densitometry analyses were normalized to GAPDH and values are presented as the mean ± SEM. ^∗^*p* < 0.05, ^∗∗^*p* < 0.01 when compared to sham control; ^∗∗∗^*p* < 0.001 when compared to sham and to 30 and 60 min; ^+^*p* < 0.05 when compared to 30 and 60 min (Bonferroni’s multiple comparisons test). Randomization: animals were distributed in groups by computer-generated randomization sequence.

**Figure 1A** shows the migration of proBDNF (37 kDa) and mBDNF (14 kDa), which summation correspond to the total BDNF, in the PFC of rats after 15, 30, or 60 min of anodal S-eDCS (400 μA for 11 min) or sham procedure applied over the left mPFC.

ANOVA showed statistically significant differences across groups from different times of measurements for total BDNF [*F*(3,15) = 8.83, *p* = 0.0023] (**Figure 1B**), proBDNF [*F*(3,15) = 6.68, *p* = 0.007] (**Figure 1C**) and mBDNF [*F*(3,15) = 37.92, *p* < 0.0001] (**Figure 1D**) isoforms.

Regarding total BDNF, smaller levels (*p* < 0.01) were observed 15 min after DC stimulation (S-eDCS) when compared to sham (S-sham), but BDNF levels from S-eDCS were almost like S-sham at 30 and 60 min after DC stimulation, which showed to be significantly larger when compared to BDNF levels at 15 min (*p* < 0.05).

The intensity of proBDNF was small after 15 min of anodal single DC stimulation, but it was clearly lower after 30 and 60 min (*p* < 0.05) when compared to S-sham (**Figure 1C**).

The levels of mBDNF in the S-eDCS group followed the pattern of the total BDNF, that is, were significantly lower at 15 min (*p* < 0.001) when compared to S-sham, being equivalent to this group at 30 and 60 min (**Figure 1D**). The levels of mBDNF were significantly higher (*p* < 0.001) in these times intervals when compared to those observed after 15 min of the anodal eDCS application (**Figure 1D**).

### Experiment 2

**FIGURE 2:**
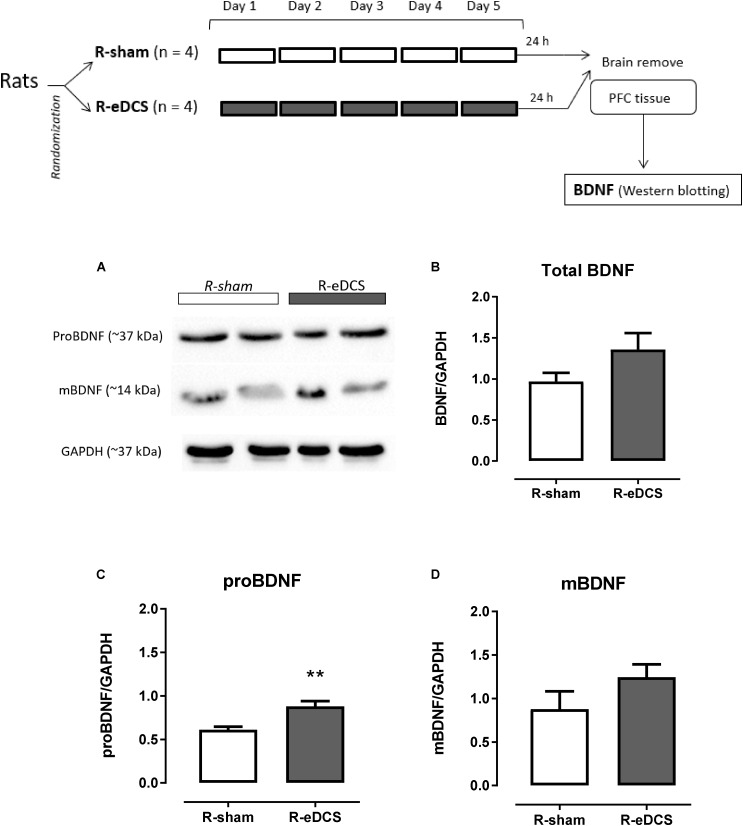
Western blot analysis of brain-derived neurotrophic factor (BDNF) in the prefrontal cortex (PFC) after five (once a day) consecutive sessions of sham (R-sham, *n* = 4) or epidural direct current stimulation (R-eDCS, *n* = 4) at 400 μA current intensity during 11 min through a 5-mm round electrode implanted over the left medial Prefrontal Cortex (mPFC). A diagram of general procedures is shown at the top. **(A)** Bands of the immunoreactivity related to the migration of the proBDNF and mBDNF shown in two animals – biological replicate – from each group; Densitometry analysis for **(B)** Total BDNF (intensity of proBDNF plus mBDNF), **(C)** proBDNF, and **(D)** mBDNF. Densitometry analyses were normalized to GAPDH and values are presented as the mean ± SEM. ^∗∗^*p* < 0.01 when compared to sham control (*t*-test for independent samples). Randomization: animals were distributed in groups by computer-generated randomization sequence.

Immunoreactive bands related to the migration of proBDNF and mBDNF in the PFC after five consecutive applications of anodal eDCS applied over the left mPFC are shown in **Figure 2A**.

When comparing the normalized intensity between R-sham and R-eDCS, levels of total BDNF and mBDNF were elevated after the repetitive anodal eDCS (**Figures 2B,D**), and the level of proBNDF [*p* < 0.01, *t*(6) = 3.89] was significantly higher in the R-eDCS when compared to R-sham (**Figure 2C**).

## Discussion

This study aimed to understand the effects of non-invasive cortical DC stimulation on BDNF expression in the PFC using identical experimental set up of our previous study showing improvement of spatial working memory in rats (de Souza Custodio et al., 2013). We observed that after a single application of eDCS, levels of total BDNF and of the mBDNF isoform were low 15 min after stimulation, but their levels were back to the control at the time intervals of 30 and 60 min, and after five consecutive sessions the BDNF expression was affected in the opposing direction.

Our results suggest that changes in the polarity of neuronal membranes induced by anodal DC may have interfered in the expression of BDNF, as well as on its post-translational modification form, i.e., mBDNF. These changes occur only in a very short period (15 min), not being detected in longer periods (30 and 60 min). Differently from these findings, we found that proBDNF levels were significantly lower at 30 and 60 min after stimulation.

In neurons, proBDNF is rapidly converted to mBDNF, which depends on proteases (Matsumoto et al., 2008). Differences in BDNF isoforms levels could be associated with alterations in the activity and/or expression of these proteases or the downstream cascade due to polarity changes induced by the anodal DC. Thus, after the short period of 15 min, anodal eDCS may have favored the processes involved in the conversion of proBDNF to its mature form. In fact, high-frequency neuronal activity seems to control the ratio of extracellular proBDNF/mBDNF by regulating the secretion of extracellular proteases, and this balance regulates the diametrically opposite functions of these two isoforms (Nagappan et al., 2009).

Twenty-four hours after the last session of the repetitive anodal eDCS applied over the mPFC, total BDNF and both isoforms levels, but especially proBDNF, were higher in the PFC of animals receiving the repetitive DC stimulation when compared to control. The high levels of proBDNF, as a precursor, would convert to mBDNF and subsequently the total BDNF would rise.

In mature brain BDNF seems to be synthetized, stored and released in a use-dependent manner (Lu, 2003). Thus, in highly demanding or increased activity conditions, such as the one induced by working memory processing or the repetitive anodal DC stimulation, the BDNF secretion, storage and release would increase and change the neuroplasticity. In fact, the anodal DC stimulation boosted synaptic plasticity by enhancing BDNF expression in response to LTP induction and learning in the hippocampus (Podda et al., 2016), and the repetitive anodal tDCS increased levels of BDNF and of NMDA receptors mRNA and the dendritic spine density on the apical dendrites of mPFC layer V pyramidal cells of diabetic rats (Wu et al., 2017).

There are limitations in this study that need to be mentioned. Although considering that the sample sizes (*n* = 4 per group) matched those reported in studies involving molecular analysis (Matsumoto et al., 2008; Blazquez et al., 2015), the present findings are based on this limited samples size. Thus, they should be considered as preliminary and need to be replicated in larger samples. Another limitation was that animals were naïve to behavioral procedures, but this study aimed to strictly examine the influence of single and repetitive anodal DC stimulation on PFC BDNF levels.

In summary, levels of BDNF in the PFC, especially proBDNF, were lower after a single exposition and higher after repetitive anodal DC stimulation applied over the left mPFC when compared to sham. Thus, changes in prefrontal BDNF levels may disclose molecular changes underlying the plasticity induced by cortical anodal DC stimulation, which may have opposite effects when applied in single or multiple sessions.

## Author Contributions

EMN-P, SGdF, CWM and JCdSC: conception and design of study, acquisition of data, and analysis and/or interpretation of data. EMN-P, SGdF, CWM, JCdSC, LCdMR, and MDMVL: drafting the manuscript, revising the manuscript critically for important intellectual content, and approval of the version of the manuscript to be published.

## Conflict of Interest Statement

The authors declare that the research was conducted in the absence of any commercial or financial relationships that could be construed as a potential conflict of interest.

## Footnotes

1 www.sbcal.org.br
